# Experimental Assessment of Nocebo Effects and Nocebo Side Effects: Definitions, Study Design, and Implications for Psychiatry and Beyond

**DOI:** 10.3389/fpsyt.2019.00396

**Published:** 2019-06-14

**Authors:** Kate Faasse, Suzanne G. Helfer, Kirsten Barnes, Ben Colagiuri, Andrew L. Geers

**Affiliations:** ^1^School of Psychology, University of New South Wales, Sydney, NSW, Australia; ^2^Department of Psychology, Adrian College, Adrian, MI, United States; ^3^School of Psychology, University of Sydney, Sydney, NSW, Australia; ^4^Department of Psychology, University of Toledo, Toledo, OH, United States

**Keywords:** nocebo effect, side effects, experimental design, research methods—psychology, no treatment control

Interest in nocebo effects is increasing exponentially: a Google Scholar search for articles referencing *nocebo* in 1998 (20 years ago) yields 90 results, increasing to 449 in 2008 (10 years ago) and to 1600 in 2018. Increased attention has likely resulted from recognition of the prevalence and potential seriousness of nocebo effects in clinical contexts. It is estimated that up to 97% of reported pharmaceutical side effects are *not* caused by the drug itself but rather by nocebo effects and symptom misattribution (1). These nocebo effects can cause symptoms serious enough to require hospitalization and medical intervention (2). As a result of the increased recognition of the importance of nocebo effects, experimental research seeking to understand how nocebo effects are formed has also intensified.

As the literature on nocebo effects has expanded, additional methodological decision points arise for researchers in this area. In this article, we discuss a set of methodological issues that result from emerging approaches to studying nocebo effects, including distinctions between designs for standard nocebo effects versus nocebo side effects, the information provided by selecting different types of control groups in experimental designs, and the distinction between “true” nocebo effects and symptom misattribution. This discussion will focus on between-subjects designs, using examples of nocebo effects that are generated by verbal/written transmission of information. For each issue, we compare the different methodologies and seek to highlight the strengths and limitations of these approaches.

## Nocebo Effects and Nocebo Side Effects Designs: Definitions

Definitions of the nocebo effect typically focus on the role of negative expectations in producing aversive outcomes [e.g., Refs. (3–7)]. In contrast, Faasse (8) extends this definition to incorporate past experience and other aspects of the treatment context. We would refine this further to define nocebo effects as unpleasant or adverse outcomes triggered by the treatment *context*, beyond any inherent pharmacological effects of the treatment itself. These nocebo effects are scientifically measurable effects caused by psychological processes including negative expectations, classical conditioning, and observational learning (9).

Although not always differentiated, there are two variants of nocebo effects that researchers examine: primary nocebo effects and nocebo side effects. Experimental designs that study *primary nocebo effects* focus on nocebo effects as the central “action” or primary negative outcome of a treatment/medical condition. Such outcomes have been described by Hahn (5, 6) as nocebo effects, where he distinguished these from what he called “placebo side effects,” whereby a treatment intended primarily for benefit can cause harmful outcomes. As an example of a primary nocebo effect—when the potential adverse outcome is framed as the primary, or focal, effect of the treatment—Benedetti and colleagues (10) informed postoperative patients that a saline injection would increase pain, resulting in elevated pain. This design contrasts those of *nocebo side effect* experiments in which participants are informed of the main (typically beneficial) outcome of a treatment/condition, and also unpleasant outcomes that are ancillary to this main outcome. This conceptualization of nocebo side effects is similar to that of Barksy and colleagues (3) as a phenomenon occurring when placebo treatment results in unpleasant side effects. For example, Neukirch and Colagiuri (11) gave participants experiencing sleep difficulty an inert treatment to improve sleep—with or without the suggestion that it created a specific side effect (increased or decreased appetite). The results revealed that participants given the side-effect warning reported more changes in appetite—in the expected direction—than those not given the side-effect warning.

Notably, recent evidence suggests that primary nocebo and nocebo side effect manipulations do not produce equivalent results. Caplandies and colleagues (12) used an experimental design that compared the two nocebo effects and found that when headache pain was described as the *primary* effect of sham transcranial direct current stimulation (tDCS), headaches were significantly more likely to occur than when headache was described as a side effect of tDCS. These results indicate that nocebo effect instructions can produce different outcomes depending on whether the adverse effect is described as the primary effect or a side effect of treatment.

## Nocebo Effects and Nocebo Side Effects: Experimental Design Selection

Making a clear distinction between a primary nocebo effect and a nocebo side effect design is valuable for several reasons. First, these two designs correspond to different clinical care circumstances. Primary nocebo effect designs relate to situations in which negative outcomes can occur without a concomitant benefit, such as when patients are given a warning about potential pain due to a medical condition (e.g., broken leg) or disease course (e.g., *rheumatoid arthritis*), which leads to increased negative expectations. In contrast, nocebo side effect designs are analogues of situations when there is a beneficial treatment that may also cause adverse effects. These designs have greater correspondence to medical treatments where treatment descriptions and instructions—including informed consent protocols, physician warnings, drug labels, and direct-to-consumer advertising—suggest that adverse outcomes can accompany a primary treatment benefit. Consequently, in the study of nocebo effects, researchers should decide between primary nocebo effect or nocebo side effect designs based on the applied circumstance they wish to understand.

A second reason for distinguishing between these two types of designs is that the pivotal mediating and moderating variables may diverge. Consider the case of moderating variables. When examining nocebo side effects, but not primary nocebo effects, variables such as number of side effects listed or their order could be critical moderators to assess. Additionally, it is possible that the contribution of different psychological mediators varies with these different designs. For example, primary nocebo effect designs emphasize adverse outcomes, whereas nocebo side effect designs emphasize the positive treatment effect as well as co-occurring adverse outcomes; because of this greater emphasis on negative outcomes, individuals in primary nocebo effect designs may devote more higher-order cognitive processes to thinking about the negative symptoms than individuals in the nocebo side effect designs (13). It should also be noted that nocebo side effects and placebo effects can, theoretically, co-exist within the same person, and the experience of benefits and unpleasant side effects may influence one another.

## Nocebo Effects and Nocebo Side Effects: Control Conditions

A second issue to consider is the appropriate control conditions for these designs. Here, we differentiate between several options.

***No-treatment control group***. It is widely accepted that when investigating the *placebo* effect, a placebo-treated group must be compared with an untreated control group in order to detect “true” rather than “perceived” placebo effects (14). The inclusion of an untreated comparison group allows researchers to distinguish between improvements *caused* by placebo administration and other factors that can result in apparent improvements, including natural history of the condition, Hawthorne effects, and regression to the mean. However, the importance of this distinction between “true” and “perceived” *nocebo* effects—and the inclusion of a no-treatment control group—is less well recognised (15).

We argue that no-treatment control groups are important for both primary nocebo and nocebo side effect designs. A simple laboratory procedure to test for true *nocebo* effects—both primary and side effects—would involve two conditions. In one condition, participants would take a sham treatment or undergo a sham procedure described as having either a primary or secondary (i.e., side effects) unpleasant outcome. In the other condition, participants would not get the nocebo treatment or procedure (i.e., they form the no-treatment control group), but undertake all other study components. An increase in the rate of unpleasant outcomes in the “treated” group would be indicative of a true nocebo effect—because comparing with the untreated control rules out natural history, Hawthorne effects, and regression to the mean, as alternative explanations.

***Sham treatment/no-information control group***. A second control condition that may be employed is one in which participants are given a treatment or procedure, but are not provided with information about possible unpleasant outcomes (primary or side effect; see **Table 1**). Thus, when a *sham treatment/no-information control group* is compared to a *sham treatment/negative information group*, participants in both conditions engage in the treatment activity (12). This procedure keeps constant factors such as naturally occurring symptoms, treatment administration, and engagement in other experimental activities across the conditions. This control can be used for either primary nocebo or nocebo side effect studies and is useful for identifying the specific effect of the information provided. Some limitations of this control condition, however, are 1) because treatments or procedures are given without corresponding information about possible side effects or adverse outcomes, it has less ecological validity than the no-treatment control condition described above, and 2) the control group does not provide information on the generation of “true” nocebo effects, i.e., the magnitude of the overall nocebo effect compared to individuals with no treatment.

**Table 1:** Examples of control and experimental conditions in nocebo research, and information provided by these comparisons.

Control condition	Experimental condition	Information provided
No treatment	Sham treatment plus negative information about adverse outcomes	Evidence of “true” nocebo effect; no information about the role of information provision and subsequent expectations, or treatment context
Sham treatment with no information about adverse outcomes	Sham treatment plus negative information about adverse outcomes	Effect of information on nocebo outcomes, controls for effect of treatment administration; no evidence for “true” nocebo effect, participants may form own expectations in the absence of information about sham treatment
Sham treatment with standard information about adverse outcomes	Sham treatment with modified experimental information (typically employed to test strategies to *reduce* nocebo effects)	Effect of modified information presentation on nocebo outcomes; no evidence for “true” nocebo effect

These two types of control conditions are not exhaustive. For example, as indicated in **Table 1**, other variations could include giving participants a treatment with “standard” or usual care information in order to test the effect of other information provision strategies, for example, standard information versus positive framing of information about adverse outcomes, which focuses on the proportion of patients who will *not* experience these unpleasant outcomes (16, 17). Of most importance, however, is that researchers consider carefully their hypotheses and relevance to clinical practice, consider which factor(s) differ between their chosen conditions, and utilize appropriate control conditions in experimental designs that will allow them to most appropriately test their research question. The control conditions described here are complementary rather than mutually exclusive, and we recommend that researchers consider including a no-treatment control as well as (for example) an information-related control condition. Finally, although this discussion has focused on designs using sham treatments, it is important to note that nocebo effects occur in response to active medical treatments too. In studies examining nocebo effects from active treatments, a no-treatment control condition is less likely to be helpful than an *active treatment/no-information control group*, which controls for the physiological effect of the treatment itself.

## Nocebo Effects and Nocebo Side Effects: Misattribution

A third consideration in experimental designs to study nocebo effects and nocebo side effects is the role of symptom misattribution. In contrast to “true” nocebo effects, “perceived” nocebo effects are those symptoms that would have occurred regardless of treatment administration but are (mistakenly) attributed to the treatment. Misattribution is particularly relevant to the study of nocebo *side* effects, such as a where a patient is experiencing regular headaches, starts a new medication, and subsequently misattributes these headaches to the treatment. Although such misattributed symptoms are undoubtedly important in how patients view their treatments and influence their health care decisions, there are likely to be different processes underlying the development of misattributed and true nocebo side effects. When designing experimental studies to investigate true nocebo side effects, assessing baseline symptoms that may be subject to later misattribution and encouraging participants to report *all* symptoms experienced regardless of perceived cause may help to assess or reduce the influence of misattribution on results. If researchers wish to explicitly study misattribution, participants who receive an experimental treatment can be asked whether they believe their symptoms were *caused* by the treatment they received.

## Summary

Nocebo effects and nocebo side effects play an important role in the outcomes of medical care. Heightened recognition of their importance and increased experimental research seeking to understand how nocebo effects are formed have raised the need for consideration of the methodological decisions that researchers face in studying the nocebo effect. These decisions include whether to examine primary nocebo effects or nocebo side effects, appropriate control conditions, and differentiating true and perceived nocebo effects. Future research would benefit from careful selection of study design and assessment of nocebo outcomes. Such steps will contribute to generating a deeper understanding of how both primary nocebo effects and nocebo side effects develop.

## Author Contributions

AG and KF contributed to the conception of the work and wrote the first draft of the manuscript. All authors critically revised the manuscript for important intellectual content and provide approval for publication of the content.

## Funding

KF is supported by an Australian Research Council Discovery Early Career Researcher Award (DE180100471); BC is supported by an Australian Research Council Discovery Early Career Researcher Award (DE160100864). The funder played no role in the conceptualization or preparation of this work. The researchers are independent from the funder.

## Conflict of Interest Statement

The authors declare that the research was conducted in the absence of any commercial or financial relationships that could be construed as a potential conflict of interest.

## References

[1] Mahr A Golmard C Pham E Iordache L Deville L Faure P Types, frequencies, and burden of nonspecific adverse events of drugs: analysis of randomized placebo-controlled clinical trials. Pharmacoepidemiol Drug Saf (2017) 26:731–41. 10.1002/pds.4169 28176407

[2] Reeves RR Ladner ME Hart RH Burke RS Nocebo effects with antidepressant clinical drug trial placebos. Gen Hosp Psychiatry (2007) 29(3):275–7. 10.1016/j.genhosppsych.2007.01.010 17484949

[3] Barsky AJ Saintfort R Rogers MP Borus JF Nonspecific medication side effects and the nocebo phenomenon. Jama (2002) 287(5):622–7. 10.1001/jama.287.5.622 11829702

[4] Colloca L Nocebo effects can make you feel pain: negative expectancies derived from features of commercial drugs elicit nocebo effects. Science (New York, N.Y.) (2017) 358(6359):44. 10.1126/science.aap8488 PMC5754642 28983038

[5] Hahn RA The nocebo phenomenon: concept, evidence, and implications for public health. Preventive Med (1997) 26:607–11. 10.1006/pmed.1996.0124 9327466

[6] Hahn RA Expectations of sickness: concept and evidence of the nocebo phenomenon. In: Kirsch I, editor. How expectancies shape experience. Washington, DC: American Psychological Association (1999). p. 333–56. 10.1037/10332-014

[7] Wager TD Atlas LY The neuroscience of placebo effects: connecting context, learning and health. Nat Rev Neurosci (2015) 16(7):403–18. 10.1038/nrn3976 PMC6013051 26087681

[8] Faasse K Nocebo effects in health psychology. Austr Psychol (2019) 1–13. 10.1111/ap.12392

[9] Colloca L Placebo, nocebo, and learning mechanisms. In: Benedetti F, editor. Placebo., vol. 225 Berlin Heidelberg: Springer-Verlag (2014). p. 17–35. 10.1007/978-3-662-44519-8 25304524

[10] Benedetti F Amanzio M Casadio C Oliaro A Maggi G Blockade of nocebo hyperalgesia by the cholecystokinin antagonist proglumide. Pain (1997) 71(2):135–40. 10.1016/S0304-3959(97)03346-0 9211474

[11] Neukirch N Colagiuri B The placebo effect, sleep difficulty, and side effects: a balanced placebo model. J Behav Med (2015) 38(2):273–83. 10.1007/s10865-014-9590-5 25119580

[12] Caplandies FC Colagiuri B Helfer SG Geers AL Effect type but not attribute framing alters nocebo headaches in an experimental paradigm. Psychol Consciousness Theor Res Pract (2017) 4(3):259–73. 10.1037/cns0000130

[13] Geers AL Briñol P Petty RE An analysis of the basic processes of formation and change of placebo expectations. Rev Gen Psychol (2018) 23:211–9. 10.1037/gpr0000171

[14] Ernst E Resch KL Concept of true and perceived placebo effects. Bmj (1995) 311(7004):551. 10.1136/bmj.311.7004.551 7663213 PMC2550609

[15] Colloca L Miller F The nocebo effect and its relevance for clinical practice. Psychosom Med (2011) 73:598–603. 10.1097/PSY.0b013e3182294a50 21862825 PMC3167012

[16] Barnes K Faasse K Geers A Helfer S Sharpe L Colloca L Can positive framing reduce nocebo side effects? Current evidence and recommendation for future research. Front Pharmacol (2019) 10:167. 10.3389/fphar.2019.00167 30894815 PMC6414427

[17] Faasse K Huynh A Pearson A Geers A Helfer S Colagiuri B The influence of side effect information framing on nocebo effects. Ann Behav Med (2018) 1–9. 10.1093/abm/kay071 30204841

